# Suicidal Behavior and Neurological Illnesses

**DOI:** 10.4172/2167-1044.S9-001

**Published:** 2013-04-18

**Authors:** Steven S Coughlin, Leo Sher

**Affiliations:** 1 Post-Deployment Health Epidemiology Program, Office of Public Health, Department of Veterans Affairs, Washington, DC, USA; 3 James J. Peters Veterans’ Affairs Medical Center, New York, USA; 4 Mount Sinai School of Medicine, New York, USA

**Keywords:** Amyotrophic lateral sclerosis, Epilepsy, Multiple sclerosis, Suicide, Veterans

## Abstract

**Objective:**

Suicidal ideation and behavior have been associated with a variety of neurological illnesses. Studies are ongoing in combat veterans and other groups to examine possible mechanisms and pathways that account for such associations.

**Method:**

This article provides a review of the literature on suicide ideation and suicidal behavior in patients with neurological illnesses including publications on veteran’s health and military medicine. Studies of suicide attempts and deaths in people with neurological illnesses are also reviewed.

**Results:**

The studies summarized in this review indicate that there are important linkages between suicidal ideation and behavior and neurological conditions, including epilepsy, multiple sclerosis, and amyotrophic lateral sclerosis.

**Conclusion:**

Additional studies are needed to further clarify why suicide ideation and suicidal behavior are associated with neurological diseases, in order to improve quality of life, alleviate patient distress, and prevent nonfatal and fatal suicide attempts in veteran and non-veteran populations.

## Introduction

Over 36,000 people in the U.S. die each year from suicide. A much larger number of people attempt suicide, but survive the experience. An estimated 25 suicide attempts occur for every fatal suicide [1]. Suicide is more frequent among men whereas non-fatal suicidal behaviors are more prevalent among women and in persons who are unmarried or have a psychiatric disorder [2]. Psychological autopsy studies have shown that more than 90% of people who commit suicide suffer from psychiatric disorders [3,4]. Demographic risk factors for suicidal ideation, developing a suicide plan, and non-fatal suicidal behaviors include being female, being younger, having less educational attainment, and being unemployed [5]. The study of suicidality and suicide prevention programs are important aspects of post-deployment health and military medicine [6]. About 20% of all known suicides in the U.S. occur among current or former military personnel [7].

Although it is widely recognized that suicide ideation and suicidal behaviors occur more frequently among persons with certain psychiatric illnesses (e.g., major depression, bipolar disorder, personality disorders, schizophrenia, substance abuse) [3,8–10], additional factors are likely to account for the transition from suicidal ideation to suicide plans or attempts [4]. For example, lack of psychosocial support from family and friends, social disadvantage, psychosocial stress due to financial problems or a disrupted relationship, and the absence of a therapeutic alliance with a clinical provider can be detrimental [1].

Previous authors have noted that suicide ideation and suicidal behaviors can be related to an individual’s physical illness and to unhealthy behaviors such as heavy alcohol consumption [11–13]. Suicidal ideation and behavior have been associated with a variety of neurological illnesses, although relatively few attempts have been made to examine possible mechanisms and pathways that account for such associations, while comparing and contrasting key pathways across various neurological conditions.

The objective of this article is to review the recent literature on suicidal ideation and behavior occurring among adults suffering from neurological illnesses including the literature on veteran’s health, including epilepsy, multiple sclerosis, and amyotrophic lateral sclerosis. The general approach used to identify references cited in this article was to identify relevant medical subject headings (MeSH) used by the National Library of Medicine and to search PubMed bibliographic databases for appropriate articles. Of particular interest were clinical and epidemiologic studies of suicidal ideation and suicidality published since 2000. Clinical descriptions of individual patients and case series involving very small numbers of patients were omitted. The bibliographic searches were limited to studies published in English. However, because suicidality and neurological illnesses are global health concerns, the bibliographic searches were not limited to studies carried out in the U.S. Additional citations were identified by reviewing systematic reviews and review articles identified through PubMed searches (for example, recent review articles on the epidemiology of suicidality). The equally important topic of suicidality and Traumatic Brain Injury (TBI), spinal cord injury, and polytrauma will be examined in a future article. In order to narrow the focus of the current review, suicide ideation and behavior occurring in Parkinson’s disease and Alzheimer disease are not considered.

## Overview of Suicidal Ideation and Suicidal Behaviors

Suicidal ideation and suicidal behaviors can be conceptualized as occurring over a continuum from suicidal ideation, development of a suicide plan, non-fatal suicide attempt(s), or death from suicide [5]. Suicides reflect only a small proportion of the total impact of suicidal behavior; substantially more persons are hospitalized as a result of nonfatal suicidal behavior than are fatally injured. An even greater number are either treated in ambulatory settings or not treated at all. Nock et al. [2] defined nonfatal suicidal thoughts and behaviors (i.e., suicidal behaviors) as suicide ideation (which refers to thoughts of engaging in behavior intended to end one’s life), suicide plan (the formulation of a specific method through which one intends to die), and suicide attempt (engagement in potentially self-injurious behavior in which there is at least some intent to die). Nonsuicidal self-injury such as self-cutting can also occur in which a person has no intent to die [2]. While suicidal behavior and nonsuicidal self-injury are distinct behaviors, nonsuicidal self-injury is a risk factor for suicide attempts and suicide. Roughly 55–85% of self-injurers have made at least one suicide attempt [14,15]. The suicide rate for patients with borderline personality disorder and nonsuicidal self-injury is about 8–10% [16,17]. Community surveys of suicide behaviors have sometimes categorized suicidal thought or behavior into 4 groups of increasing severity: suicide ideation only, suicide plan, unplanned suicide attempt and planned suicide attempt [18]. The Department of Veterans Affairs recently adopted the U.S. Centers for Disease Control and Prevention’s Self-Directed Violence Classification System nomenclature to facilitate research and epidemiologic surveillance efforts [19].

Retrospective studies suggest that suicidal ideation fluctuates and that the process that people may go through before they attempt suicide is complex [20]. Suicidal thoughts and behaviours tend to be transient in nature, coming and going repeatedly over time [18]. Mathias et al. [21] noted that suicidal ideation should be thought of as a state that would be expected to fluctuate in intensity over time rather than as a psychological trait. Suicide attempters are distinguished from nonattempters by specific traits and by a family history of suicidal behavior. Suicidal behavior is usually associated with mood and anxiety disorders, borderline and antisocial personality disorders, alcohol and drug dependence, and greater lifetime aggression and impulsivity in attempters than in nonattempters [22–24]. A further issue is that some differences have been noted between the characteristics of persons who attempt suicide and those who complete suicide [25].

The risk of nonfatal self-injury (both suicidal and nonsuicidal in nature) rises significantly in the U.S. during adolescence and young adulthood and then decreases monotonically throughout adulthood [2]. Rates of non-fatal self-injury are higher among females than in males, in contrast to a pattern of higher suicide rates among males. Only a small percentage of people who attempt suicide eventually complete suicide, although suicide attempts are an indicator of extreme psychological distress and an important predictor of eventual completed suicide [5]. In fact, the best predictor of suicide is a history of a previous suicide attempt. It has also been observed that more severe methods of attempted suicide confer greater risk of completed suicide [26].

Risk factors for attempted suicide include suicide ideation, developing a suicide plan, prior suicide attempts, hopelessness, having a positive family history of suicide or psychiatric illness, and recent loss of an important relationship [1]. Suicide ideation and suicidality have been related to an individual’s psychiatric or physical illness [2,11]. In over 90% of suicides, this fatal outcome is a complication of a psychiatric disorder. Sixty percent of all suicide victims have a mood disorder at the time of death [22,27–29]. Personally knowing somebody who completed or attempted suicide is associated with an increased risk of experiencing suicidal ideation or attempting suicide, especially among young peole [20,30]. Other risk factors for suicide attempts and death from suicide include lack of social support, unemployment, and low income [31]. Social disadvantage, financial distress, and legal difficulties have been identified as risk factors for suicidal ideation and precipitants of suicidal acts [20,32,33].

The lifetime prevalence of suicide ideation has been estimated to be 5.6–14.3 percent [2]. The 12-month prevalence of suicide ideation has been estimated to be 2.1–10.0 percent. The variation in rates is likely due, in part, to variability in the methods used to assess suicidal behaviors [2]. Bossarte et al. [34] recently examined the prevalence of suicide ideation among active military and veteran participants in a national health survey in the U.S. The prevalence of suicide ideation and attempts identified in their study were similar to published estimates of those outcomes in the U.S. general population, 3.7% and 0.5%, respectively. In a study of Operation Enduring Freedom/Operation Iraqi Freedom (OEF/OIF) veterans from Connecticutt, Pietrzak et al. [35] found that 12.5% (34 of 272) of the respondents reported contemplating suicide in the 2 weeks prior to completing the survey. The veterans who reported suicidal ideation were more likely to screen positive for post-traumatic stress disorder (PTSD), depression, or problem drinking [35]. They also scored higher on measures of psychosocial difficulties, stigma, and barriers to care, and lower on measures of resilience and social support. Other studies have shown that suicidal ideation is associated with combat exposure [36]. Among the highest published rates of suicidal ideation (63%) are those from a study of homeless men and women [37].

### Ideation as a risk factor for suicide attempt or death

Persons who experience suicidal ideation, especially those who have comorbid conditions such as major depression, bipolar disorder, alcohol abuse or dependence, or chronic pain, have a higher risk of nonfatal or fatal suicide attempt [38–40]. Suicidal ideation has been associated with increased risk of suicidal behavior in community-based studies [12,20,40] and in follow-up studies of patients seen for psychiatric care [31,41]. Combat veterans are more likely to have suicidal ideation (often associated with PTSD and depression) and they may be more likely to act on a suicidal plan [42]. A recent study by Britton et al. [38] identified suicidal ideation as a warning sign for suicide among U.S. veterans who died within 1 week of receiving healthcare from the Veterans Health Administration (VHA).

There is likely to be variability in the extent to which suicidal ideation predicts suicide in clinical and community samples. In a 12-year follow-up study of a community sample in Baltimore, Maryland, Kuo et al. [40] found that persons who reported suicidal ideation at baseline were more likely to report having attempted suicide at follow-up (relative risk=6.09, 95% confidence interval 2.58–14.36). In a longitudinal study in a community sample, adolescents who had suicidal ideation at age 15 were more likely to have attempted suicide by age 30 [33]. An estimated 34 percent of persons who experience suicidal ideation go on to make a suicide plan and about 72 percent of persons with a suicide plan go on to make a suicide attempt [2,43]. An estimated 26 percent of persons who experience suicidal ideation make an unplanned suicide attempt.

A variety of preventive factors have been identified that can help protect people with suicidal ideation from attempting suicide. These include family responsibilities and child-related concerns, fear of suicide, fear of social disapproval, and life-affirming cultural values [1]. Concern shown by others has been inversely associated with suicidal ideation [44]. Psychosocial support from family, friends, and community, and having a sense of purpose, have been reported to protect against suicidal behavior among OEF/OIF veterans who may have PTSD [35]. Skills in problem-solving and having a therapeutic alliance with a clinical provider can also be important [1]. Resilience to suicidal behavior is influenced by genetic factors, parenting, psychiatric and medical illnesses, especially affecting the brain, e.g., epilepsy, migraine, Huntington’s disease, alcoholism and substance abuse [22,27,45–49]. The relationships may be in different directions; genetic factors may both increase or decrease resilience to suicidality; illnesses affecting the brain can increase suicide risk. Important suicide protectors include effective clinical care for physical and psychiatric disorders (including substance abuse); easy access to a variety of clinical interventions and support for help seeking; restricted access to highly lethal means of suicide; strong connections to family and community support, and cultural and religious beliefs that discourage suicide and support self preservation [50].

### Assessment

The identification of people who are having thoughts of suicide or of harming themselves in some way, and ensuring their access to mental health care services and appropriate follow-up, has been highlighted in the psychiatric, medical, and public health literature [7]. Suicidal ideation can be a highly distressing experience and is a valid target for clinical intervention [51]. Such ideation can be passive, fleeting, intermittent, active, and intense, with or without the intent to die [1]. As part of clinical evaluation and suicide risk assessment, suicidal ideation can be distinguished from suicidal intent. The latter is the subjective expectation and desire to die by a self-destructive act [1].

Suicide assessments consider such factors as previous suicide attempts, mood and anxiety symptoms, coexisting psychiatric disorders including alcohol and drug abuse, the role of any medications (stimulants, antidepressants, other), and the lack of family and social support. The presence of a suicide plan or the possibility of implementing a suicide plan is also considered.

Studies of suicide assessment tools such as the Scale for Suicidal Ideation and the Beck Hopelessness Scale have shown associations between higher scores and death by suicide [7]. The utility of such tools in routine screening in primary care practice is uncertain because of the potential for false positive and false negative identification of persons at risk of suicide and because bush primary care practitioners do not have time to administer these or other scales [7]. Suicide risk assessments performed as part of psychiatric care are determinations made for the purpose of treatment and patient management and are not long-term predictions of suicide risk [1]. Suicide risk assessment has a quasiquantitative dimension in that risk of suicide increases with various combinations of risk factors [1].

## Epilepsy

Epilepsy affects about 3 million Americans. About 200,000 new cases of epilepsy and seizures occur each year in the U.S. A complex relationship exists between epilepsy and suicidality [52,53]. Epilepsy both increases the risk of the development of psychiatric conditions (for example, depression) and the risk of suicide among people with psychiatric disorders [52]. In addition to psychiatric illness, risk factors for suicide among people with epilepsy include temporal-lobe epilepsy, female sex, and the onset of epilepsy at an earlier age [52]. People with epilepsy have a higher risk of suicide ideation and suicidal behaviors than the general population [54]. Suicide risk increases soon after the onset of epilepsy and then gradually decreases [52]. Onset of epilepsy in the first 17 years of life is associated with a significant increase in risk for suicide compared with those whose epilepsy started at an age of more than 29 years [53]. The relation is one of mutual causation since injuries sustained during a suicide attempt can injure the brain and cause epileptic seizures. A further issue is that epilepsy and suicidal ideation may share common neurobiological mechanisms such as disturbances of neurotransmitters [54]. For example, serotonergic disturbances are present in both epilepsy and suicidal behavior. Antiepileptic drugs have been associated with increased risk of suicidality by some regulatory agencies, although the finding has not been supported by some studies and meta-analyses [52,55–57]. The U.S. Food and Drug Administration requires that labeling of antiepileptic drugs include a warning and that a medication guide be provided to patients, informing them of the risks of suicidal thoughts or actions. The existence of a causal association between antiepileptic medications and suicidality has been a topic of continuing scientific discussion and debate. One reason for the elevated risk of suicide in people with epilepsy might be social disadvantages and stigma associated with epilepsy.

Post-traumatic forms of epilepsy are especially important in combat veterans who are more likely to experience mild, moderate, or severe traumatic brain injuries, PTSD, and major depression than other veterans [58–60]. Epileptic seizures, PTSD, depression and suicide ideation and attempts are more frequent among persons with traumatic brain injury [58,59,61]. People with epilepsy should be regularly evaluated for depression and suicidality by their physician, and when depression or suicidality is present, therapeutic interventions are necessary.

## Multiple Sclerosis

Multiple sclerosis (MS) is a chronic neurological disease with a variable clinical course [62]. The pathophysiological mechanisms include neuronal/axonal damage and loss, demyelination, inflammation, remyelination and repair, and gliosis; the latter refers to a proliferation of astrocytes in damaged areas of the central nervous system [62]. The axonal loss and gliosis that occurs in MS can result in a progression of disability. The disability experienced by MS patients varies widely. The overall life expectancy for MS patients has been estimated to be 6–7 years less than that of persons without the disease [63]. Estimates of the annual incidence of MS vary by country, sex, race, and history of military service [64–66]. Women have a higher risk of MS than men. Among U.S. Armed Forces personnel from 2000–2009, the annual incidence rate was 12.9 per 100,000 person-years [67]. Black non-Hispanics had a higher incidence rate (18.3 per 100,000 person-years) than white non-Hispanics (12.5 per 100,000 person-years) [67]. Because many MS patients do not die from their chronic illness, estimates of the prevalence of MS are much higher. For example, Noonan et al. [68] found that the prevalence of MS in 3 US communities ranged from 47.2–109.5 per 100,000 persons. In a recent study from France the prevalence of MS was estimated to be between 170.9–188.2 cases per 100,000 persons [69]. Epidemiologic and genetic studies have shown that MS likely results from a complex interaction between genetic susceptibility and environmental factors [70]. Genetic studies have confirmed that the immune system plays a key role in the development of MS [71].

Depression is common in patients with MS and other neurodegenerative diseases [72–75]. Paparrigopoulos et al. [76] postulated that depression occurring in MS may be due to a complex interplay of biological, disease-related, behavioral, and psychosocial factors. The neuroinflammatory reaction is involved in the pathophysiology of MS. Immune sensitization to neural antigens could develop within the systemic compartment consequent to exposure to cross-reacting peptides plays an important role in the neurobiology of MS [73,74]. The etiology of depression in MS patients may be due to a psychological reaction to the diagnosis or associated disability, to neuroanatomical or neurochemical changes associated with neurodegeneration, or to psychiatric side effects of corticosteroids or interferons used to treat MS [77–79]. Other factors may contribute to depression among MS patients include maladaptive coping strategies, loss of recreational opportunities, having poor quality relationships, and high levels of stress and fatigue [80]. In evaluating depression occurring in patients with a neurodegenerative disease such as MS, providers should draw a distinction between the syndrome of major depression (as defined by the Diagnostic and Statistical Manual of Mental Disorders, 4^th^ revision) and symptoms of MS itself [72]. The lifetime prevalence of major depression in MS patients may approach 50% [72].

Population-based studies have generally shown that people with MS have about a two-fold increased risk of suicide, after adjustment for age [81–83], although no significant association was observed between suicide and MS in a study conducted in Finland between 1964 and 1993 (standardized mortality ratio=1.7, 95% confidence interval 0.9–2.7) [84]. Suicide risk may be particularly high in the first year following the diagnosis of MS, and among young men [81]. A recent study found that 3.7% (15 of 404) of MS patients in Denmark had attempted suicide; the rate was not significantly different than that of the general population of the County of Funen, Denmark [85].

Over a quarter of MS patients may contemplate suicide [72,86]. In a clinic-based study of 140 MS patients in Toronto, Canada, Feinstein [86] found that 28.6% (40 of 140) of patients had thought about committing suicide and 6.4% (9 of 140) had attempted suicide. Those with a history of suicidal intent were more likely to have a history of major depression than the non-suicidal group (83% versus 18%). Factors associated with suicidal intent among MS patients included living alone, having a positive family history of mental illness, reporting more social stress, and having a lifetime history of major depression, anxiety disorder, or alcohol abuse [86]. Turner et al. [39] studied suicidal ideation in 445 VHA patients with MS by linking computerized medical records with information obtained via a mail survey. Suicidal ideation was assessed using the PHQ suicide item. About 29.4% (131 of 445) of respondents had suicidal ideation and 7.9% (35 of 445) had persistent suicidal ideation (more than half the days) over the past 2 weeks. In bivariate analyses, suicidal ideation was associated with younger age, earlier disease course, progressive disease subtype, lower income, not being married, having less social support, not driving, having higher levels of physical disability, and depression [39].

Previous authors have noted that providers who care for MS patients should closely monitor their patients for depression and suicide ideation, that MS patients with suicidal ideation should be referred for immediate psychiatric consultation, and that depression in MS patients can be effectively treated with antidepressant medication or psychotherapy [72,76,78,87]. Current and emerging MS therapies that may show promise across the spectrum of disease have recently been reviewed by Castro-Borrero et al. [88] and Bomprezzi et al. [89]. It is important to make sure that new therapies will not be associated with suicidality.

## Amyotrophic Lateral Sclerosis

Amyotrophic lateral sclerosis (ALS) is a devastating disease characterized by progressive loss of motor neurons [90]. In the U.S., the prevalence of ALS is about 5 cases per 100,000 persons, with somewhat higher rates among men. Most people with ALS are between the ages of 40 and 70. About 5% of ALS cases are familial [91]. Considerable evidence from both patients and animal models indicates impairment of all neurovascular unit components including the blood-brain and blood-spinal cord barriers [92]. The neurodegeneration seen in ALS can occur within the corticospinal tracts, brain stem neuclei, and spinal cord anterior horns [93]. The neurodegeneration can extend beyond motor cerebral neurons into the frontal and temporal lobes although frank dementia is rare [93]. Patients often experience weakness of limb, abdominal, thoracic, or bulbar muscles. The latter are muscles of the mouth and throat that are responsible for speech and swallowing. The one relatively effective drug for ALS, Riluzole, a medication that preferentially blocks tetrodotoxin-sensitive sodium channels which reduces influx of calcium ions and probably prefents stimulation of glutamate receptors [94], prolongs survival for up to 6 months [95,96]. Currently, most therapeutic efforts for ALS patients are centered on symptom management, including such important areas as respiration, nutrition, secretions, communication, physical therapy and exercise, muscle cramps and spasticity, pain, and depression [97]. Although ALS patients often die within 3 to 5 years from diagnosis about 10% of patients survive for more than 10 years [98].

Palmieri et al. [99] examined suicidal ideation in 42 patients with ALS. The Rorschach test was used in the assessments. The investigators found that suicidal ideation was more frequent in recently diagnosed ALS patients [99]. A population-based study in Sweden identified 6,642 patients with incident ALS between 1965 and 2004 using the Swedish Inpatient Register [90]. The investigators found an almost 6-fold increased risk for suicide among ALS patients (standardized mortality ratio 5.8, 95% confidence interval 3.6–8.8). Patients who committed suicide were on average about 7 years younger at the time of their first hospitalization as compared with those who did not commit suicide.

It is important to note that ALS is associated with cognitive impairments [100]. Cognitive abnormalities are associated with suicidal behavior [101,102]. Therefore, cognitive impairments may mediate the effect of ALS on suicidality.

In a study by Rabkin et al. [103] of 80 patients with late-stage ALS, about 8–9% of patients had major depression at the time of any visit. The frequency of depression did not generally increase as death approached [103]. More severe depressive symptoms and greater hopelessness were correlated with higher scores on measures of suffering, anger, perceived caregiver burden, and weariness [103]. During the study, 14 patients received antidepressant medication and 6 saw a counselor or therapist [103]. The authors noted that depression affects only a small subset of patients with late-stage ALS and that the overall results from their study provide a “picture of resiliency rather than despair” [103]. Lule et al. [104] conducted two studies of depression and quality of life among ALS patients. One study was longitudinal in design and the other compared ALS patients with healthy controls. About 28% of the patients had signs of depression based upon the ALS Depression Inventory; the severity of depression was inversely related to educational status [104]. Depressive symptoms were not found to be correlated with extent of physical impairment or time since diagnosis [104]. A measure of quality of life was similar among ALS patients and healthy controls, indicating that ALS patients can experience a satisfactory quality of life without manifestations of depression even if they are severely physically impaired [104]. Additional studies of the psychological health of patients with ALS have also found that clinically significant depression is not as frequent or severe as might be expected (based upon studies of other life-threatening medical illnesses), although the psychological impact of ALS on patients, family, and caregivers is profound [105,106]. Several studies have shown that ALS patients often report surprisingly high subjective quality of life despite their marked physical impairment [104,107].

## Summary and Conclusions

The results of this literature review highlight the importance of an awareness of the psychological distress experienced by patients with neurological conditions such as, epilepsy, MS, and ALS. Scientific understanding of the psychoneurobiological mechanisms and pathways that account for suicidal ideation and suicidal behaviors in patients with neurological disorders is still in its infancy. Measures of executive dysfunction have been associated with suicidal behavior in some neuropsychological studies of research participants with a history of suicidal behavior and healthy controls [108]. Twin-studies and other family studies have provided evidence of a heritable risk of suicidal behavior [2,109–115]. Much of the family history of suicidal behavior may be explained by the risk associated with psychiatric conditions such as major depression, bipolar illness, and schizophrenia.

Advances in proteomics, genomics, and metabolomics, and ongoing and planned clinical trials for neurorestoration within the human central nervous system, offer hope for patients suffering from MS, ALS, and for their family members [98,116–118].

Researchers have examined specific psychological constructs that may explain why neuropsychiatric disorders are associated with suicidal behaviors. These constructs include the presence of hopelessness, anhedonia, impulsiveness, and high emotional reactivity [2]. A variety of psychological and psychoanalytic models and theories of suicidal behaviors have been proposed. For example, in the Interpersonal Theory of Suicide, interpersonal connectedness is posited to be a protective factor for suicidal behavior [119]. The presence of a psychiatric disorder is a consistently reported risk factor for suicidal ideation and behavior [2].

Physical illnesses and chronic pain are important risk factors for suicidal ideation and behaviors. Epilepsy can occur during childhood, adolescence, and young adult-hood, or later in life, whereas neurodegenerative diseases such as Parkinson’s disease, stroke, Alzheimer disease, and dementia-which were outside the scope of the current review—are primarily illnesses that afflict the elderly. Multiple sclerosis and ALS may also occur in the elderly and in middle-aged persons. In people of all ages, physical illnesses, depression, and loss of important relationships are important risk factors for suicidal behaviors. Primary care providers can play an important role in suicide prevention among higher-risk patients including the elderly [2]. In a study by Isometsa et al. [29], 41% of older adults had seen their primary care physician within 28 days of committing suicide. A meta-analysis of 40 studies found that almost 45% of people of various ages who committed suicide had contact with primary care providers within 1 month of the suicide [120]. The availability of effective clinical care for physical, mental, and substance abuse disorders is important for the prevention of suicide behaviors. Bruffaerts et al. [18] noted that the help seeking process of suicidal people is complex. People experiencing suicidal ideation often feel pessimistic and hopeless and they may not have positive expectations that treatment will be helpful for them [18]. Once someone has decided to seek treatment, they may wait and see whether the problem subsides by itself and whether they can “ride out the storm.” Adherence with recommended treatments is also important. A variety of strategies have been proposed to encourage people experiencing suicidal ideation or intent to seek help, to provide referrals, and to improve referral follow-through and attendance [18,121,122]. In the U.S., these strategies include a national network of suicide prevention crisis lines, including the VA national crisis line (veteranscrisisline.net), the National Suicide Prevention Lifeline (http://www.suicidepreventionlifeline.org), and comparable resources for U.S. military personnel. A congressionally mandated comprehensive VA suicide prevention program began in 2007 [123]. National strategies for suicide prevention are periodically updated as new scientific information becomes available.
